# Synthesis of Heterostructured TiO_2_ Nanopores/Nanotubes by Anodizing at High Voltages

**DOI:** 10.3390/ma17133347

**Published:** 2024-07-06

**Authors:** Ta Quoc Tuan, Le Van Toan, Vuong-Hung Pham

**Affiliations:** 1 School of Materials Science and Engineering, Hanoi University of Science and Technology (HUST), 01 Dai Co Viet, Hanoi 100000, Vietnam; tuan.taquoc@hust.edu.vn (T.Q.T.); levantoan2011@gmail.com (L.V.T.); 2 Laboratory of Biomedical Materials, Hanoi University of Science and Technology (HUST), 01 Dai Co Viet, Hanoi 100000, Vietnam; 3 Department of Environmental Engineering, Faculty of Physics and Chemical Engineering, Le Quy Don Technical University, 236 Hoang Quoc Viet Road, Hanoi 100000, Vietnam

**Keywords:** biomaterials, corrosion rate, anodizing, cell attachment

## Abstract

This paper reports on the coating of heterostructured TiO_2_ nanopores/nanotubes on Ti substrates by anodizing at high voltages to design surfaces for biomedical implants. As the anodized voltage from 50 V to 350 V was applied, the microstructure of the coating shifted from regular TiO_2_ nanotubes to heterostructured TiO_2_ nanopores/nanotubes. In addition, the dimension of the heterostructured TiO_2_ nanopores/nanotubes was a function of voltage. The electrochemical characteristics of TiO_2_ nanotubes and heterostructured TiO_2_ nanopores/nanotubes were evaluated in simulated body fluid (SBF) solution. The creation of heterostructured TiO_2_ nanopores/nanotubes on Ti substrates resulted in a significant increase in BHK cell attachment compared to that of the Ti substrates and the TiO_2_ nanotubes.

## 1. Introduction

Titanium (Ti) and its alloys have long been known as widely used materials in the biomedical field thanks to their physical and mechanical properties, such as high strength, good corrosion resistance, low modulus elasticity, high hardness, and biocompatibility [1,2]. The biocompatibility of Ti is attributed to the formation of a TiO_2_ film on its surface [3,4]. However, this naturally occurring 1.5–10 nm TiO_2_ layer exhibits inherent instability and poses limitations for practical implant applications due to its inadequate cell adhesion properties [5,6]. In the field of implants, porous surfaces are also of particular interest because they provide a high binding site for cellular growth [7,8]. Therefore, there have been several attempts to address the TiO_2_ coating with a high surface area. The first approach uses a combination of acid etching with anodizing [9,10] or milling with anodizing [11,12]. Another method uses the anodizing of porous Ti substrates [13,14]. All the mentioned methods can produce both a protective coating and a high binding site for the cellular growth of Ti implants.

Anodizing the surface of titanium at high voltages creates a titanium surface with many valuable properties, such as enhanced corrosion protection for the titanium substrate. Jeremiasz Koper et al. conducted anodization of titanium in a solution mixture of H_3_PO_4_ and HF at voltages ranging from 30 V to 240 V to improve the corrosion resistance of titanium, with a focus on biomedical applications [15].

Warittha Asumpinwong et al. also conducted research on the anodization of titanium alloy in a H_3_PO_4_ solution on Ti-6Al-4V alloy substrates at voltages ranging from 100 to 300 V at room temperature to enhance the corrosion resistance of the titanium alloy [16].

Il Song Park et al. conducted a study on the anodization of pure titanium in a solution composed of glycerophosphate (disodium salt: monohydrate) and calcium acetate, within a voltage range of 220 V to 340 V, to create an adhesive layer for hydroxyapatite [17].

In addition, numerous studies have investigated the anodization of titanium and its alloys in H_3_PO_4_ and H_2_SO_4_ solutions within a voltage range of 100 V to 250 V to impart color to titanium for decorative and biomedical implant applications [18,19,20].

The temperature of the electrolyte solution significantly influences the structure of the titanium oxide layer during anodization due to its impact on the solution’s conductivity, consequently affecting the voltage and/or current density throughout the anodization process [21,22,23].

However, these processes are lengthy, which may impose limitations on their practical applications. Therefore, in this study, we have developed a simple and cost-effective method for synthesizing heterostructured TiO_2_ nanopores/nanotubes, coating Ti substrates with high corrosion resistance. This was achieved through the anodization method conducted at high voltages ranging from 50 to 350 V in a hybrid solution comprising ethylene glycol, ammonium fluoride, and water.

At present, the literature which delineates the intricate mechanism underlying titanium anodization at elevated voltages is scant. Nevertheless, we hypothesize that during anodization at elevated voltages, the anode surface undergoes electron transfer reactions, resulting in the formation of various titanium cations with different oxidation states, such as Ti^2+^, Ti^3+^, Ti^4+^, etc. Additionally, at the electrodes, water oxidation and reduction processes occur as follows:

Anode: 2H_2_O → O_2_ + 4H^+^ + 4e^−^; (R1)

Cathode: 2H_2_O + 2e^−^ → H_2_ + 2OH^−^. (R2)

Under the influence of the applied voltage, hydroxide ions (OH^−^) migrate to the anode where they react with titanium cations (Ti^x+^):

Ti^x+^ + xOH^−^ = Ti(OH)_x_. (R3)

These Ti(OH)_x_ compounds aggregate and precipitate onto the titanium surface. Moreover, Ti^4+^ also interacts with other O^2−^ ions to form a layer of TiO_2_ with excellent corrosion-resistance properties, closely adhering to the substrate.

To our knowledge, there are no reports on the synthesis of heterostructured TiO_2_ nanopores/nanotubes by one step of anodizing. The effect of the anodizing voltages on the evolution of heterostructured TiO_2_ nanopores/nanotubes will be addressed. Confocal laser-scanning microscope results showed that the heterostructured TiO_2_ nanopores/nanotubes have better cell attachment than those of the Ti substrates and the TiO_2_ nanotubes.

## 2. Experimental Procedure

### 2.1. Anodizing Titanium with High Voltage

This study received approval from the Institutional Review Board of Hanoi University of Science and Technology (381/QĐ-ĐHBK-QLNC). Heterostructured TiO_2_ nanopores/nanotubes were formed on Ti substrate using an anodizing method at different voltages up to 350 V to tailor the microstructure of the coatings. The Ti substrate had dimensions 10 × 10 × 1 mm^3^ and had been ground with sandpaper of 1000 grit. The ground Ti substrates were then washed under ultrasonic conditions and with distilled water to remove contamination. Prior to the coating of heterostructured TiO_2_ nanopores/nanotubes, Ti substrate was used as an anode and Pt was used as a cathode. The electrolytes were prepared using ammonium fluoride (NH_4_F, Sigma, St. Louis, MO, USA, 99.9%), ethylene glycol solution (C_2_H_6_O_2_, Sigma, 99.9%), and H_2_O. The anodizing was carried out at different voltages from 50 V to 350 V for 1 h using a ITECH Auto Range DC power supply (IT6723G 600V/5A/850W, ITECH Electronics, Nanjing, China).

### 2.2. Surface Morphology Analysis and Electrochemical Characteristics

The microstructure of the coating was characterized by scanning electron microscopy (SEM) (JEOL, JSM-6700F, JEOL Techniques, Tokyo, Japan). The phase of the heterostructured TiO_2_ was determined using X-ray diffraction (Bruker, Berlin, Germany) analysis with an X-ray diffractometer CuKα1 radiation λ = 1.5406 Å.

The electrochemical measurements were conducted using the Zahner Zennium Pro device (Kronach, Germany), controlled by Thales Z2.10 USB software, in a standard three-electrode setup. Specifically, the counter electrode (CE) was a platinum electrode, the working electrode (WE) was a titanium electrode anodized at different voltages between 50 V and 150 V, and the reference electrode (RE) was a saturated calomel electrode.

Prior to corrosion testing, the samples were immersed in SBF solution for 30 min to stabilize them. Tafel plots were generated by linear potential scanning from −100 mV to +100 mV, with a scan rate of 10 mV/s. The polarization resistance in the Tafel extrapolation method was calculated using Formula (1):

(1) $$R_{p}=\frac{\beta_{a}\times \beta_{c}}{2.303\times i_{corr}\times (\beta_{a}+\beta_{c})}$$

where the anodic and cathodic Tafel slopes of the sample are denoted as β_a_ and β_c_, respectively. The corrosion current density of the substrate is represented by i_corr_, while R_p_ stands for the polarization resistance of the substrate.

The corrosion rate (v) (g/m^2^h) can be calculated from the corrosion current density using Faraday’s law:

(2) $$v=\frac{M}{nF}\times i_{corr}=3.73\times 10^{-4}\times \frac{M}{n}\times i_{corr}$$

where M is the molar mass of the metal (g/mol), n is the number of electrons exchanged per metal atom, and F is the Faraday constant.

Equation (3) is applied to evaluate the corrosion protection effectiveness of TiO_2_ nanotubes and heterostructured TiO_2_ nanopores/nanotubes in comparison to bare Ti in SBF solution using the Tafel curve:

(3) $$H\left(\%\right)=\frac{{CR}_{TiO_{2}}-{CR}_{Ti}}{{CR}_{Ti}}\times 100\%$$

where H (%) represents the corrosion protection efficiency of TiO_2_, while ${CR}_{TiO_{2}}$ stands for the corrosion rate of TiO_2_ layers (mg/m^2^h). ${CR}_{Ti}$ refers to the corrosion rate of bare Ti (mg/m^2^h).

### 2.3. Biological Compatibility Evaluation

Cell attachment was evaluated with a confocal laser microscope (FV3000RS, Olympus, Nagano, Japan) after cell culturing on the surface for 48 h. The baby hamster kidney (BHK) cells were maintained in DMEM (Gibco, Paisley, UK) supplemented with 10% FBS (Gibco) and 1% streptomycin (Gibco). Before the in vitro cell tests, the Ti substrate and TiO_2_ nanopores/nanotubes were sterilized by autoclaving at 121 °C for 60 min. The same concentrations of cells and volumes were seeded on the bare Ti and TiO_2_ nanopores/nanotubes. The BHK cells on the nanopores/nanotubes and the Ti substrate were fixed in 4% paraformaldehyde in PBS for 7 min, washed in PBS, permeabilized with 0.1% Triton X-100 in PBS for 7 min, washed in PBS, and stained with fluorescent phalloidin (Invitrogen, Waltham, MA, USA) for 60 min. The cell nuclei were labeled with DAPI (Himedia, Mumbai, India) for 10 min. The stained cells adhered to the nanopores/nanotubes and the Ti substrate was subsequently placed on a glass coverslip; cell attachment was observed at various magnifications on two observation channels, HSD1 and HSD2, corresponding to the DAPI and Alexa Fluor 555 dyes with emission wavelengths of 461 nm and 568 nm, respectively.

## 3. Results and Discussion

### 3.1. Surface Properties

**Figure 1:**
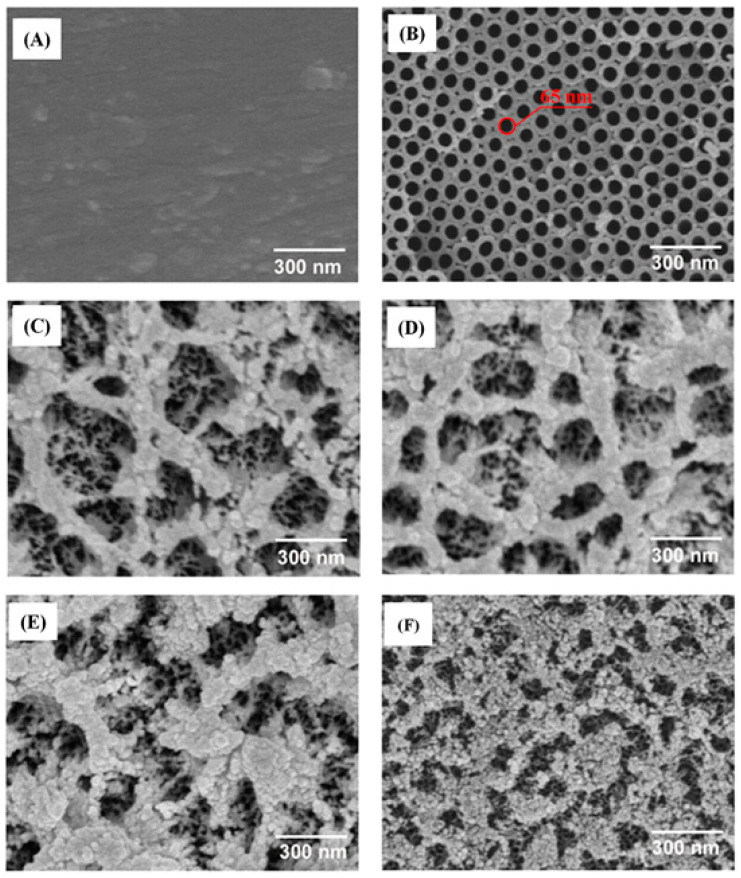
FE-SEM images of Ti synthesis by anodizing at different voltages: (**A**) Ti substrates, (**B**) 50 V, (**C**) 100 V, (**D**) 150 V, (**E**) 250 V, and (**F**) 350 V.

Figure 1A–F show FE–SEM images illustrating the morphological variations of Ti and TiO_2_ nanostructures synthesized via anodization at different applied voltages. The Ti substrates exhibited a smooth microstructure (Figure 1A). Anodization at 50 V (Figure 1B) resulted in well-defined TiO_2_ nanotubes with an inner diameter of 65 nm, a characteristic observed in previous studies [24,25]. At 100 V (Figure 1C), a heterostructure comprising TiO_2_ nanopores and nanotubes emerged, with nanopore diameters around 500 nm and tube diameters of approximately 20 nm. Increasing the voltage to 150 V (Figure 1D) and 250 V (Figure 1E) while maintaining tube diameters at approximately 20 nm led to reductions in nanopore diameters to 30 nm and 20 nm, respectively. At 350 V (Figure 1F), TiO_2_ nanopores/nanotubes transformed into structured TiO_2_ nanowalls/nanotubes while retaining a tube diameter of 20 nm.

**Figure 2:**
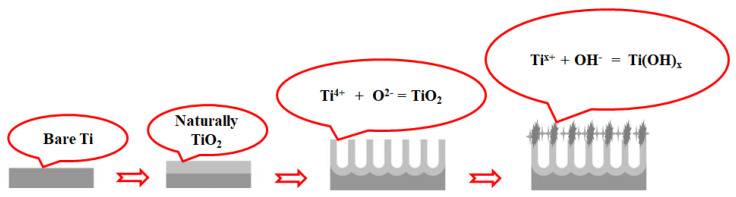
Schematic of heterostructured TiO_2_ nanopore/nanotube synthesis by anodizing at high voltages.

The formation of TiO_2_ nanotubes at different voltages is elucidated in Figure 2. At a voltage of 50 V, TiO_2_ compound formation occurs via the ion mechanism, facilitated by the interaction between Ti^4+^ and O^2−^ ions. The initiation of the TiO_2_ nanotube formation originates from the Ti substrates [26,27]. Additionally, Ti surfaces retain Ti^3+^ and Ti^2+^ cations due to the Ti oxidation process. Under the influence of high voltage, these cations readily bind with OH^−^ anions generated from water electrolysis, yielding Ti(OH)_x_ compounds via reaction (R4).

Ti^x+^ + xOH^−^ = Ti(OH)_x_ (x = 2; 3; 4) (R4)

The Ti(OH)_x_ compound adheres to the surface of the TiO_2_ nanotubes. Therefore, in Figure 1C–E, a relatively distinct surface layer can be observed on the nanotube surface, while the TiO_2_ nanotubes undergo noticeable thinning due to the dissolution process induced by F^−^ anions, as depicted in reaction (R5). When the voltage increases to 350 V, the Ti(OH)_x_ layer becomes less discernible, as at this voltage the dissolution of the TiO_2_ nanotubes via reaction (R5) takes precedence over the formation of the Ti(OH)_x_ compounds as described in reaction (R4).

TiO_2_ + 6F^−^ + 2H_2_O = TiF_6_^2−^ + 4OH^−^ (R5)

Based on the FE-SEM results obtained, we selected samples of both the TiO_2_ nanotubes at 50 V and the heterostructured TiO_2_ nanopores/nanotubes at 150 V for further evaluation in subsequent experiments.

### 3.2. X-ray Diffraction Study

**Figure 3:**
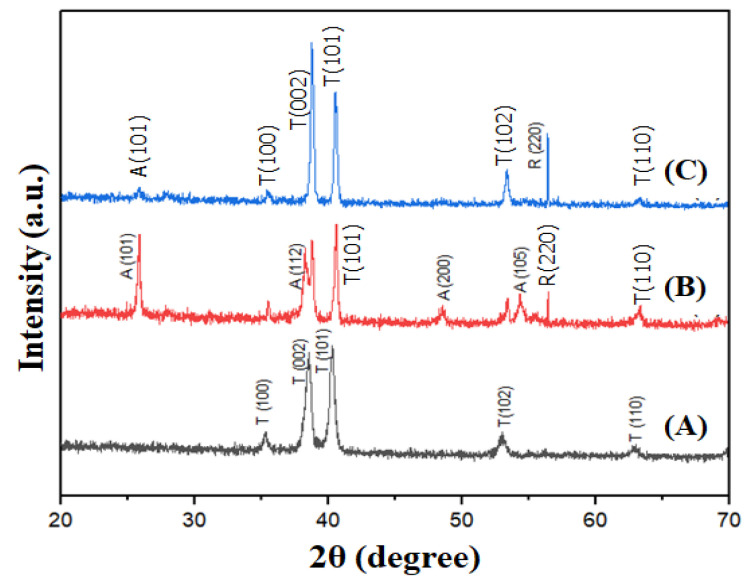
The X-ray diffraction patterns of (**A**) pure Ti; (**B**) TiO_2_ 50 V; and (**C**) TiO_2_ 150 V, all heat-treated at 550 °C.

To ascertain the structural characteristics, phase composition, and crystalline quality of the fabricated materials, we conducted X-ray diffraction (XRD) analysis of the samples post heat treatment at 550 °C, as depicted in Figure 3. Figure 3A illustrates the XRD spectrum of bare Ti, while Figure 3B,C represent samples of the TiO_2_ nanotubes at 50 V and the heterostructured TiO_2_ nanopores/nanotubes at 150 V, respectively. Notably, Figure 3B exhibits distinct diffraction peaks around 2θ angles of approximately 25.7°, 38.3°, 48.64°, and 54.37°, corresponding to the (101), (112), (200), and (105) crystal planes characteristic of TiO_2_ with an anatase phase structure, according to the JCPDS card 21-1272 standard. In contrast, in Figure 3C, the intensity of these peaks significantly diminishes, with the emergence of the prominent diffraction peak (220) of TiO_2_ with a rutile phase structure at a 2θ angle of approximately 56.4°, according to the JCPDS card 21-1276 standard. 

On the surface of titanium, there always exists a thin oxide barrier layer, which is relatively mechanically robust and chemically inert; however, this oxide layer typically does not contain anatase (101) [23]. During anodization at low voltages, phases of anatase (such as (112), (200), and (105)) are observed. Conversely, during anodization at high voltages, these phases are often absent due to the potentially significant alterations in the structure and properties of the oxide barrier layer induced by high-voltage anodization [15]. This can lead to the formation of alternative forms of titanium oxide, such as rutile or other impure forms, rather than anatase. Additionally, the conditions of high voltage can create harsh reaction environments, which are unfavorable for the formation and maintenance of anatase crystal faces [28].

Notably, the presence of Ti(OH)_x_ compounds was not observed in this X-ray diffraction due to their thermal instability. At 550 °C, Ti(OH)_x_ compounds undergo thermal decomposition into TiO_2_ and H_2_O, as described in reaction (R6).

Ti(OH)_4_ = TiO_2_ + 2H_2_O (R6)

### 3.3. Electrochemical Properties

**Figure 4:**
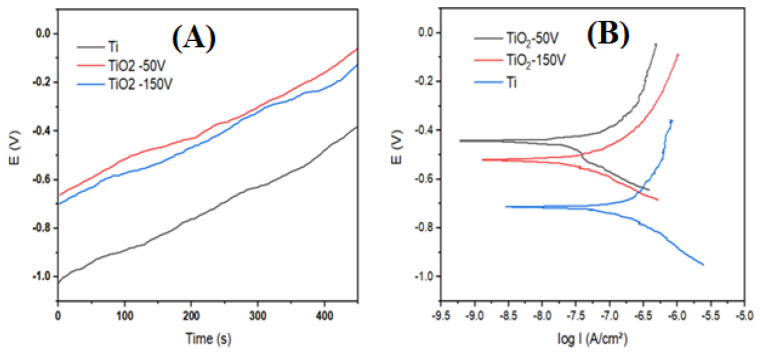
(**A**) Open circuit potential plots (OCP); (**B**) Tafel curves.

The electrochemical characteristics of the Ti samples after anodization are showed in the open circuit potential (OCP) plots and the Tafel curves in Figure 4; the corresponding parameters are outlined in Table 1. It is evident that the corrosion potential (E_corr_) of the TiO_2_ nanotubes and the heterostructured TiO_2_ nanopores/nanotubes is more positive compared to the untreated Ti sample. For the bare Ti, E_corr_ = −0.717 V; after anodization at 50 V, the corrosion potential of the TiO_2_ nanotubes increases to E_corr_ = −0.442 V; and after the anodization at 150 V, the corrosion potential of the heterostructured TiO_2_ nanopores/nanotubes is E_corr_ = −0.514 V. This indicates that the TiO_2_ nanotubes and heterostructured TiO_2_ nanopores/nanotubes become more chemically inert. Parameters such as I_corr_ and R_corr_ also demonstrate that after anodization the TiO_2_ nanotubes and heterostructured TiO_2_ nanopores/nanotubes exhibit lower corrosion rates compared to the untreated Ti sample, suggesting the beneficial contribution of the TiO_2_ coating in forming a durable protective layer. This can be explained by the formation of a high-resistance TiO_2_ layer on the titanium surface after anodization, leading to a reduced conductivity and charge-transfer capability, thereby diminishing the chemical activity of the sample. The corrosion protection efficiency of the TiO_2_ nanotubes at 50 V is 87.78%, higher than the heterostructured TiO_2_ nanopores/nanotubes at 150 V, which achieve a value of 76.26%. This difference arises because the surface of the heterostructured TiO_2_ nanopores/nanotubes after anodization at 150 V is significantly more porous compared to the TiO_2_ nanotubes at 50 V, so the corrosion resistance of the heterostructured TiO_2_ nanopores/nanotubes at 150 V (R_corr_ = 945 Ω) is lower compared to the TiO_2_ nanotubes at 50 V (Rcorr = 1059 Ω), indicating a higher chemical activity of TiO_2_ on the heterostructured TiO_2_ nanopores/nanotubes.

**Figure 5:**
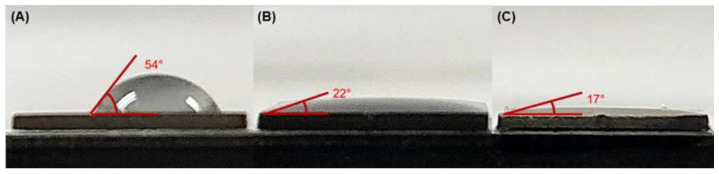
The wetting angles of (**A**) bare Ti; (**B**) TiO_2_ 50 V; and (**C**) TiO_2_ 150 V.

The porosity of the TiO_2_ layer after anodization significantly affects its wettability. Bare Ti samples exhibit poor wettability with a contact angle of approximately 54°. However, after the anodization process, the wettability of the TiO_2_ nanopores/nanotubes dramatically increases. The heterostructured TiO_2_ nanopores/nanotubes at 150 V display a more porous structure, leading to enhanced wettability, with a contact angle of approximately 17° (see Figure 5C). This makes the sample anodized at 150 V more wettable compared to the sample anodized at 50 V, which has a contact angle of approximately 22° (see Figure 5B).

### 3.4. Cell Attachment Testing

**Figure 6:**
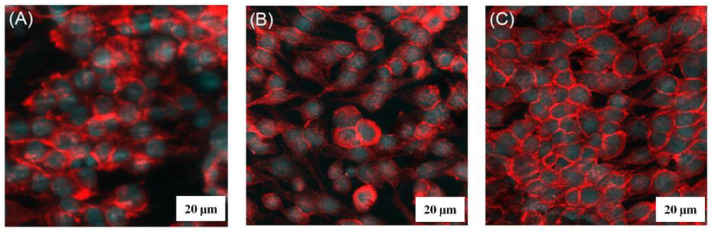
BHK cell attachment on (**A**) Ti substrates, (**B**) TiO_2_ nanotubes, and (**C**) heterostructured TiO_2_ nanopores/nanotubes.

In vitro BHK cell attachment of the heterostructured TiO_2_ nanopores/nanotubes was evaluated using a confocal laser-scanning microscope. The typical cell attachment on Ti substrates, TiO_2_ nanotubes, and TiO_2_ nanopores/nanotubes is shown in Figure 6A–C. Compared to the Ti substrates, higher cell attachment was observed for the TiO_2_ nanotubes and the TiO_2_ nanopores/nanotubes for the same cell-seeding and culturing time. Among the three surfaces, the TiO_2_ nanopores/nanotubes show the best cell attachment, demonstrating the effectiveness of the high binding site for the cell’s growth by creating heterostructured TiO_2_ nanopores/nanotubes. This result is also consistent with previous research findings [29,30,31]; the surface of titanium after anodization will form a coating with a porous nanostructure in the form of nanotubes/nanopores and heterostructures, enhancing cell adhesion and thus increasing biocompatibility.

## 4. Conclusions

In summary, we have demonstrated that heterostructured TiO_2_ nanopores/nanotubes can be successfully synthesized by anodizing Ti substrates at high voltages. Specifically, the microstructure of TiO_2_ transformed from nanotubes to heterostructured TiO_2_ nanopores/nanotubes when voltages ranging from 50 V to 350 V were applied. The diameter of TiO_2_ nanopores/nanotubes exhibited voltage dependence. The corrosion protection efficiency after the anodization of TiO_2_ nanotubes and heterostructured TiO_2_ nanopores/nanotubes dramatically increased compared to bare Ti. BHK cell attachment on the heterostructured TiO_2_ nanopores/nanotubes was significantly higher than that on Ti substrates and even TiO_2_ nanotubes.

## Figures and Tables

**Table 1:** Electrochemical characteristic parameters.

Samples	E_corr_ (V)	i_corr_ (10^−7^ A/cm^2^)	R_corr_ (kΩcm^2^)	Corrosion Rate (mg/m^2^h)	CP Efficient H (%)
Bare Ti	−0.717	1.280	332	5.73	0%
TiO_2_ 50 V	−0.442	0.157	1059	0.70	87.78%
TiO_2_ 150 V	−0.515	0.305	945	1.36	76.26%

## Data Availability

Data are contained within the article.

## References

[1] Manivasagam V.K., Popat K.C. (2021). Hydrothermally treated titanium surfaces for enhanced osteogenic differentiation of adipose derived stem cells. Mater. Sci. Eng. C.

[2] Yang Y., Zhang H., Komasa S., Morimoto Y., Sekino T., Kawazoe T., Okazaki J. (2021). UV/ozone irradiation manipulates immune response for antibacterial activity and bone regeneration on titanium. Mater. Sci. Eng. C.

[3] Cao Y., Desai T.A. (2020). TiO_2_-Based Nanotopographical Cues Attenuate the Restenotic Phenotype in Primary Human Vascular Endothelial and Smooth Muscle Cells. ACS Biomater. Sci. Eng..

[4] Ocampo R.A., Echeverry-Rendon M., Robledo S., Echeverría F.E. (2022). Effect of TiO_2_ nanotubes size, heat treatment, and UV irradiation on osteoblast behavior. Mater. Chem. Phys..

[5] Motola M., Capek J., Zazpe R., Bacova J., Hromadko L., Bruckova L., Ng S., Handl J., Spotz Z., Knotek P. (2020). Thin TiO_2_ Coatings by ALD Enhance the Cell Growth on TiO_2_ Nanotubular and Flat Substrates. ACS Appl. Bio Mater..

[6] Demetrescu I., Pirvu C., Mitran V. (2010). Effect of nano-topographical features of Ti/TiO_2_ electrode surface on cell response and electrochemical stability in artificial saliva. Bioelectrochemistry.

[7] Jung H.D., Yook S.W., Kim H.E., Koh Y.H. (2009). Fabrication of titanium scaffolds with porosity and pore size gradients by sequential freeze casting. Mater. Lett..

[8] Pham V.H., Jang T.S., Jung H.D., Kim H.E., Koh Y.H. (2012). Creation of nanoporous tantalum (Ta)-incorporated titanium (Ti) surface onto Ti implants by sputtering of Ta in Ar under extremely high negative substrate biases. J. Mater. Chem..

[9] Xiao J., Zhou H., Zhao L., Sun Y., Guan S., Liu B., Kong L. (2011). The effect of hierarchical micro/nanosurface titanium implant on osseointegration in ovariectomized sheep. Osteoporos. Int..

[10] Kim M.H., Park K., Choi K.H., Kim S.H., Kim S.E., Jeong C.M., Huh J.B. (2015). Cell adhesion and in vivo osseointegration of sandblasted/acid etched/anodized dental implants. Int. J. Mol. Sci..

[11] Wang G., Wan Y., Ren B., Liu Z. (2019). Bioactivity of micropatterned TiO_2_ nanotubes fabricated by micro-milling and anodic oxidation. Mater. Sci. Eng. C.

[12] Wan Y., Wang T., Wang Z., Jin Y., Liu Z. (2018). Construction and characterization of micro/nano-topography on titanium alloy formed by micro-milling and anodic oxidation. Int. J. Adv. Manuf. Technol..

[13] Fan X., Feng B., Liu Z., Tan J., Zhi W., Lu X., Wang J., Weng J. (2012). Fabrication of TiO_2_ nanotubes on porous titanium scaffold and biocompatibility evaluation in vitro and in vivo. J. Biomed. Mater. Res. Part A.

[14] Lee J.H., Kim H.E., Shin K.H., Koh Y.H. (2010). Improving the strength and biocompatibility of porous titanium scaffolds by creating elongated pores coated with a bioactive, nanoporous TiO_2_ layer. Mater. Lett..

[15] Jeremiasz K., Jaroslaw J. (2015). Effect of the High Voltage Anodic Oxidation on the Titanium Corrosion Resistance. Solid State Phenom..

[16] Asumpinwong W., Kanokwan S., Viritpon S. (2015). Different constant voltages of anodization on the corrosion behavior of Ti-6Al-4V alloy. Chiang Mai J. Sci..

[17] Il S.P., Tae G.W., Min H.L., Seung G.A. (2006). Effects of anodizing voltage on the anodized and hydrothermally treated titanium surface. Met. Mater. Int..

[18] Chayanis V., Tachakorn K., Pimduen R., Pisaisit C. (2023). Color Formation on Titanium Surface Treated by Anodization and the Surface Characteristics: A Review. CM Dent. J..

[19] Lucas A.G., Andrea G.S., Emilio J.P., Wido H.S., Silvia M.C., Josefina B. (2012). Chemical and mechanical properties of anodized cp-titanium in NH_4_H_2_PO_4_/NH_4_F media for biomedical applications. Surf. Coat. Technol..

[20] Giuseppe N., Marco P., Tiziana V., Andrea D.S. (2018). Colouring titanium alloys by anodic oxidation. Metalurgija.

[21] Bradford J.P., Hernandez-Moreno G., Pillai R.R., Hernandez-Nichols A.L., Thomas V. (2024). Low-Temperature Plasmas Improving Chemical and Cellular Properties of Poly (Ether Ether Ketone) Biomaterial for Biomineralization. Materials.

[22] Tian G., Dong L., Wei C., Huang J., He H., Shao J. (2006). Investigation on microstructure and optical properties of titanium dioxide coatings annealed at various temperature. Opt. Mater..

[23] Mohan L., Dennis C., Padmapriya N., Anandan C., Rajendran N. (2020). Effect of Electrolyte Temperature and Anodization Time on Formation of TiO_2_ Nanotubes for Biomedical Applications. Mater. Today Commun..

[24] Ali G., Kim H.J., Kim J.J., Cho S.O. (2014). Formation of self-organized Zircaloy-4 oxide nanotubes in organic viscous electrolyte via anodization. Nanoscale Res. Lett..

[25] Guan D., Wang Y. (2012). Synthesis and growth mechanism of multilayer TiO_2_ nanotube arrays. Nanoscale.

[26] Shokuhfar T., Sinha-Ray S., Sukotjo C., Yarin A.L. (2013). Intercalation of anti-inflammatory drug molecules within TiO_2_ nanotubes. RSC Adv..

[27] Lee K., Mazare A., Schmuki P. (2014). One-Dimensional Titanium Dioxide Nanomaterials: Nanotubes. Chem. Rev..

[28] Xue Q.G., Annabella S. (2005). Reactivity of Anatase TiO_2_ Nanoparticles: The Role of the Minority (001) Surface. J. Phys. Chem. B.

[29] Khorasani A.M., Goldberg M., Doeven E.H., Littlefair G. (2015). Titanium in Biomedical Applications-Properties and Fabrication: A Review. J. Biomater. Tissue Eng..

[30] Jiang P., Zhang Y., Hu R., Shi B., Zhang L., Huang Q., Yang Y., Tang P., Lin C. (2023). Advanced surface engineering of titanium materials for biomedical applications: From static modification to dynamic responsive regulation. Bioact. Mater..

[31] Mutalik C., Hsiao Y.-C., Chang Y.-H., Krisnawati D.I., Alimansur M., Jazidie A., Nuh M., Chang C.-C., Wang D.-Y., Kuo T.-R. (2020). High UV-Vis-NIR Light-Induced Antibacterial Activity by Heterostructured TiO_2_-FeS_2_ Nanocomposites. Int. J. Nanomed..

